# The association of depression and postoperative delirium: we may need more information

**DOI:** 10.1093/icvts/ivac228

**Published:** 2022-09-01

**Authors:** Carlos A Mestres, Eduard Quintana

**Affiliations:** Department of Cardiothoracic Surgery, University of the Free State, Bloemfontein, South Africa; Department of Cardiovascular Surgery, Hospital Clinic, University of Barcelona, Barcelona, Spain

**Keywords:** Cardiac surgery, Depression, Postoperative delirium

Cerebral complications after cardiac surgery with cardiopulmonary bypass have a substantial impact on outcomes and resource utilization. Roach *et al.* [1] defined 2 types of outcomes in a seminal contribution. Type I, death due to stroke/hypoxic encephalopathy, non-fatal stroke, transient ischaemic attack or stupor or coma at discharge; Type II, new deterioration in intellectual function, confusion, agitation, disorientation, memory deficit or seizure without evidence of focal injury [1].

Delirium is an acute organ dysfunction with mental status changes including alterations in level of consciousness, known to burden postoperative outcomes [2] and investigated in patients undergoing non-cardiac [3] and cardiac surgery [4]. Preoperative cognitive status and depression have been associated with postoperative delirium (POD). Assessment scores can be influenced by age, education or ethnicity [4]. Some strategies are currently being investigated aiming at predicting and treating POD after in different settings [5, 6].

In this issue of the Journal, Falk *et al.* [7] aimed at investigating preoperative depression as predictor of POD after cardiac surgery, analysing 1133 patients operated between 2013 and 2016. Fourteen per cent reported depressive symptoms preoperatively; the observed incidence of POD was 26%, being highest among elderly patients. One-third of patients with depression developed POD in comparison one-quarter of the non-depressed. Odds of POD were 2.19 times higher in individuals with depressive symptoms compared to controls. The onset of delirium was most common on the first 2 days after surgery. The authors concluded that in their unique population-based study, preoperative depression is associated with POD in a large proportion of patients. The corollary is that improved preoperative screening for depression and enhanced clinical surveillance in the early postoperative period for all patients is required. Although this study established the actual observed rates of POD in relation to preoperative symptoms of depression in a given population like that of the authors, there are some unclear aspects.

This study was conducted between 2013 and 2016, an acute study as authors only investigated POD rates with no follow-up. It is somewhat surprising that data have been reported after a substantially long period of time, considering as said, that this looked as an acute study. Population changes of interest may have occurred during this period. One may argue that the authors’ regional population remains stable over time and that changes might be unlikely. Although population stability may be the key factor, the readership would have been keen to integrate a brief elaboration.

The authors distributed the local version of the Patient Health Questionnaire (PHQ-9) 2 weeks before a scheduled operation. As confirmed by the authors in their revisions, 30% of their patients are operated as ‘urgent’ patients; however, in the final sample, urgent surgery ranged between 12% and 14%. The definition of ‘urgent’, patients operated within 24 h does not fully match the internationally agreed definition of urgency and may eventually fall in the category ‘emergency’. The main question would be if the PHQ-9 questionnaire is appropriately administered as the interpretation of depressive symptoms facing an elective operation may not be the same as when a patient has to be operated ‘urgently’, assuming that a number of patients requiring an urgent operation may be in shock, intubated or present with stroke, etc. Why enrolling all patients instead of only elective patients is unclear, too. Overall, 45% of the patients completed the preoperative questionnaire.

There were 3 age groups based on an administrative government age assignment of a ‘pension’ status, which may eventually only work at the authors’ place. The use of administrative databases or decisions is a matter of controversy in clinical research.

The main goal of the authors was to explore the association between depression and POD in patients undergoing cardiac surgery. This may be fine; however, there is no information other than the observed delirium rate of 26%. The authors have not elaborated on how delirium was treated and for how long the delirium status lasted or if this status was present at discharge. There is simply no follow-up and it is not easy to understand if delirium had actual negative impact on this population. No information is given with regards to actual observed mortality in this surgical cohort and if possible perioperative deaths might have been related to POD.

This is important as authors confirm in their introduction that POD is a serious complication with patients having poorer long-term outcomes, increased length of hospital stay and reduced quality of life after surgery. Although one may argue that the aim of the study was very restrictive, to explore the association between depression and POD in this surgical population, the information produced by the authors is missing and therefore, these introductory remarks are somewhat difficult to interpret them. There is no chance to know if their delirium patients had longer length of stay, had worse in-hospital and follow-up outcomes or if their quality of life after surgery was better. The prevalence of delirium and an eventual association with depression alone might be an interesting information locally but delivering additional relevant information would have enhanced the actual quality of this interesting report.

Delirium was mostly diagnosed within 48 h after the operation, in line with similar studies. This is definitely not a surprise. This study has not delved into possible organic causes of delirium like brain embolization and the readership must assume that everything observed after the operation is necessarily related with some sort of preoperative depression with, as per the information produced by the authors, multifactorial pattern including important issues like patients living alone (around one-third) or chronic alcohol consumption, moderate or higher (over 80%). These factors are not exclusive of cardiac surgical patients and may need a more accurate analysis of given societal behavioural patterns.

Data from this analysis concur with recent contributions reporting similar POD observed rates [8], in this case of patients undergoing aortic valve replacement. Data produced by Messé *et al.* [8] are substantially more powerful. Crude data may be important, however, a more granular analysis would have favoured understanding. Furthermore, the authors’ conclusions on preoperative screening should probably be expanded towards an earlier public health intervention in social behaviour as confirmed by the findings reported.

## Data Availability

The authors declare that the data collected was gathered from available databases.
